# The Role of Local Prostate and Metastasis-Directed Radiotherapy in the Treatment of Oligometastatic Prostate Cancer

**DOI:** 10.3390/cancers16183159

**Published:** 2024-09-14

**Authors:** Seo Hee Choi, Seung-Hoon Beom, Young Deuk Choi, Won Sik Ham, Hyunho Han, Woong Kyu Han, Won Sik Jang, Seung Hwan Lee, Jaeho Cho

**Affiliations:** 1 Department of Radiation Oncology, Yonsei Cancer Center, Heavy Ion Therapy Research Institute, Yonsei University College of Medicine, Seoul 03722, Republic of Korea; clickby_s@yuhs.ac; 2 Department of Internal Medicine, Division of Medical Oncology, Yonsei University College of Medicine, Seoul 03722, Republic of Korea; 3 Department of Urology, Yonsei University College of Medicine, Seoul 03722, Republic of Korea

**Keywords:** oligometastasis, radiotherapy, prostate cancer, survival outcomes, metastasis-directed radiotherapy

## Abstract

**Simple Summary:**

This study investigates the optimal timing and effectiveness of radiotherapy in treating oligometastatic prostate cancer (OMPC), which is characterized by limited metastatic spread. We evaluated the impact of early intervention with radiotherapy, targeting both the primary tumor and metastatic sites, on patient survival. Our results show that early radiotherapy to oligometastatic sites, particularly when combined with primary tumor treatment, significantly enhances survival outcomes. These findings underscore the critical importance of timely and personalized treatment strategies in improving the management and care of patients with OMPC.

**Abstract:**

**Background/Objectives:** Oligometastatic prostate cancer (OMPC) represents an early stage of metastatic disease characterized by a limited number of lesions. Recent advancements in imaging and treatment have revived interest in personalized therapies, including metastasis-directed radiotherapy (OMDRT) and primary prostate radiotherapy (PPR). This study evaluates the impact of OMDRT timing and the role of PPR on survival outcomes in OMPC patients; **Methods:** In this retrospective cohort study, 82 patients with OMPC who underwent OMDRT between 2010 and 2019 were analyzed. Patients were classified based on OMDRT timing (early vs. late) and disease type (synchronous vs. metachronous). Progression-free survival (PFS) and overall survival (OS) were the primary endpoints, assessed via Kaplan-Meier analysis and Cox proportional hazards models; **Results:** Among the patients, 36 (43.9%) had synchronous and 46 (56.1%) had metachronous OMD. With a median follow-up of 32 months, the 5-year PFS and OS rates were 77.5% and 88.5%, respectively. Early OMDRT significantly improved PFS (HR 0.461, 95% CI: 0.257–0.826, *p* = 0.009) and OS (HR 0.219, 95% CI: 0.080–0.603, *p* = 0.003). Subgroup analysis showed the most favorable outcomes for synchronous OMD patients receiving early OMDRT, with a median PFS of 22.2 months and a 5-year survival rate of 42.1%. The treatment of the primary prostate provided a survival benefit in the OS of synchronous OMD patients (5-year 83.1% vs. 50%, *p* = 0.025), and there was a further improvement in OS after PPR (5-year 87.7% vs. 50%, *p* = 0.015). **Conclusions:** Early OMDRT significantly enhances survival outcomes in OMPC, in both synchronous and metachronous cases. The integration of PPR can further improve results, emphasizing the importance of early intervention and personalized treatment strategies. To more definitively clarify our findings across various clinical situations, further studies with larger cohorts or prospective designs are necessary.

## 1. Introduction

Prostate cancer is the second most common malignancy and a leading cause of cancer-related mortality among men worldwide [1,2]. An estimated 10 to 20% of prostate cancer cases present with metastasis, a figure that has been increasing in recent years [3,4]. Oligometastatic prostate cancer (OMPC), characterized by a limited number of metastatic lesions [5,6], has garnered significant attention due to advancements in imaging that enable earlier detection. This condition represents an early stage of metastatic progression and has prompted discussions on the best therapeutic approaches. While systemic therapies such as androgen deprivation therapy (ADT) remain foundational in treating metastatic prostate cancer, there is a growing interest in personalized approaches for oligometastatic disease. In particular, localized interventions, including surgery and radiotherapy (RT) targeting both the primary tumor and metastatic sites, are being extensively researched, with multiple clinical trials currently underway.

Managing both the primary tumor and metastatic sites in OMPC can reduce tumor cell circulation and potentially induce an abscopal effect through immunomodulation, leading to systemic anti-tumor responses [7,8]. Targeting the primary tumor may also impede intratumoral adaptations and delay the onset of castration resistance [9,10]. Both surgery and RT are viable treatment options; RT not only reduces tumor burden within the irradiated area but may also induce broader anti-tumor effects. The application of RT in OMPC typically involves two main strategies: (1) targeting the primary tumor and (2) treating metastatic lesions. Clinical studies [11,12,13,14,15] suggest that primary prostate radiotherapy (PPR) in OMPC may confer survival benefits. Additionally, metastasis-directed RT, often involving stereotactic body radiation therapy (SBRT) to all metastatic sites following the radical treatment of the primary tumor, can slow further metastatic progression and delay the initiation of ADT [16,17,18,19]. Current clinical guidelines [20,21] recommend PPR for de novo oligometastatic castration-sensitive prostate cancer and metastasis-directed RT for oligorecurrent prostate cancer.

Metastasis-directed RT is gaining recognition as a promising treatment strategy for OMPC. However, further research is necessary, particularly concerning the optimal timing of metastasis-directed RT and its interaction with PPR in different clinical scenarios. The current literature lacks detailed analyses addressing these factors within real-world clinical contexts. Consequently, this study aims to assess the impact of metastasis-directed RT on survival outcomes in patients with OMPC, focusing specifically on the timing of RT and the role of PPR. By providing a comprehensive analysis of these treatment strategies, we aim to contribute to the optimization of therapeutic approaches for OMPC, ultimately enhancing clinical decision-making and patient care in this rapidly evolving field.

## 2. Methods

### 2.1. Study Design and Patient Selection

This retrospective cohort study was conducted at Yonsei Cancer Center and included 82 patients diagnosed with OMPC between 2010 and 2019. Patients were selected based on the presence of oligometastatic disease (OMD), defined as five or fewer metastatic lesions detectable through imaging modalities such as CT, MRI, or whole-body bone scans. OMD was further classified as either synchronous (metastases present at the time of initial prostate cancer diagnosis) or metachronous (metastases that developed after initial treatment for prostate cancer) [22]. All patients underwent metastasis-directed RT targeting either all or part of the detected OMD lesions (referred to as “OMDRT”). The study adhered to the Declaration of Helsinki and was approved by the Institutional Review Board (IRB) of Yonsei Cancer Center (IRB No. 4-2021-1640).

### 2.2. Treatments

At our institution, the standard initial management for OMPC typically involved hormone therapy. However, OMDRT was considered case-by-case, depending on individual patient circumstances and physician discretion. Due to evolving institutional guidelines during the study period, the timing of OMDRT varied. Some patients received OMDRT immediately upon detection of OMD (referred to as “early OMDRT”), while others received OMDRT upon the progression of OMD lesions or the onset of symptoms despite ongoing hormone therapy (referred to as “late OMDRT”). In patients with synchronous OMD, OMDRT was sometimes administered concurrently with PPR. In other cases, only OMDRT was performed without concurrent PPR. All patients with metachronous OMD had previously undergone prostate surgery and/or RT during their initial treatment phase. Based on the timing of OMDRT and the type of OMD, patients were categorized into four distinct subgroups: (1) Synchronous OMD with early OMDRT, (2) Synchronous OMD with late OMDRT, (3) Metachronous OMD with early OMDRT, and (4) Metachronous OMD with late OMDRT. Data collection included detailed clinical and treatment histories, including patient demographics, tumor characteristics, treatment modalities, and follow-up outcomes. This information was retrieved from electronic medical records and our institutional registry.

### 2.3. Statistical Analysis

The primary endpoints of the study were progression-free survival (PFS) and overall survival (OS) following OMDRT. PFS was defined as the time from the end of OMDRT to the first recorded evidence of disease progression or death from any cause, whichever occurred first. OS was defined as the time from the end of OMDRT to death from any cause. Secondary endpoints included PFS and OS from the time of initial prostate cancer diagnosis. These dual endpoints were selected to control for potential bias introduced by varying OMDRT initiation times, enabling a more precise evaluation of OMDRT’s direct impact on survival outcomes.

Kaplan-Meier survival curves were generated to estimate PFS and OS rates. Differences in survival outcomes between subgroups were assessed using the log-rank test. Additionally, multivariate Cox proportional hazards regression models were employed to adjust for potential confounders, focusing on factors that were significant in the univariate analysis. Hazard ratios (HR) and 95% confidence intervals (CI) were calculated to identify independent predictors of survival outcomes. Subgroup analyses were conducted to evaluate the impact of OMDRT timing (early vs. late) and the type of OMD (synchronous vs. metachronous) on PFS and OS. In addition, the differences in PFS and OS based on whether the primary prostate was treated and whether primary surgery or PPR was performed before or concurrently with OMDRT were also analyzed. Statistical significance was defined as a two-sided *p*-value of <0.05. All statistical analyses were performed using SPSS software (version 25.0, IBM Corporation, Armonk, NY, USA).

## 3. Results

### 3.1. Patient Characteristics

**Figure 1:**
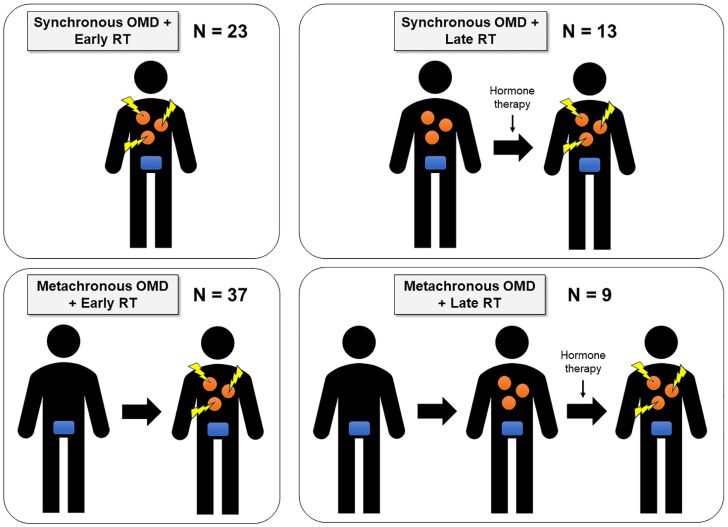
Patient subgroups by type of OMD and timing of OMDRT in this study. The orange figure represents OMD lesions, and the blue figure represents the primary prostate tumor. The yellow figure indicates that radiotherapy is being administered to each OMD lesion.

This study included 82 patients with OMPC who were treated between 2010 and 2019. The median age at diagnosis was 66 years (range, 48–83 years). Of these patients, 36 (43.9%) presented with synchronous OMD, while 46 (56.1%) developed metachronous OMD. In the synchronous OMD cohort, 23 patients (63.9%) received early OMDRT, while the remaining 13 (36.1%) received late OMDRT. Notably, 34 patients (94.4%) in this group underwent primary prostate treatment, including surgery (n = 19) or PPR (n = 15), either concurrently with or before OMDRT. Specifically, 11 patients received early OMDRT combined with PPR, while 4 received late OMDRT combined with PPR. In the metachronous OMD cohort, 37 patients (80.4%) received early OMDRT, while 9 (19.6%) underwent late OMDRT. All patients in this cohort had previously undergone prostate surgery and/or RT at initial diagnosis, with 23 patients receiving surgery (including 17 who also received salvage RT and 1 who received postoperative RT) and 23 receiving PPR. Based on the timing of OMDRT and the type of OMD, patients were categorized into four distinct subgroups: (1) Synchronous OMD with early OMDRT, (2) Synchronous OMD with late OMDRT, (3) Metachronous OMD with early OMDRT, and (4) Metachronous OMD with late OMDRT. The distribution of patients across these subgroups is illustrated in Figure 1.

In the entire cohort, 76 patients (92.7%) received OMDRT targeting all detected OMD lesions, while 6 (7.3%) received OMDRT for only a subset of the lesions. The most common site of OMD was bone (87.8%), with 68 patients having bone as the sole metastatic site. Additional metastatic sites included non-regional lymph nodes (n = 9), lung (n = 3), adrenal gland (n = 1), and local recurrence (n = 1). Regarding the radiation dose scheme for OMDRT, conventional fractionation was selected for only one patient. At the same time, moderate hypofractionation was chosen for 64 patients (78%), and ultra-hypofractionation (dose per fraction ≥5 Gy, also known as SBRT) was selected for 17 patients (20.7%). Hormone therapy was administered to 95.1% of the patients following OMD diagnosis, with 64 patients (78%) receiving it concurrently with OMDRT. Detailed patient demographics and baseline characteristics are summarized in Table 1. Additionally, there were no statistically significant differences in baseline characteristics between the subgroups, except for PSA level at the time of OMDRT (Supplementary Table S1).

### 3.2. Survival Outcomes

The median follow-up duration was 32 months (range, 6–98 months). During this period, 58 patients (70.7%) experienced disease progression after OMDRT. Recurrence at the OMDRT-treated sites was observed in 11 patients. The patterns of disease progression were categorized as follows: recurrent OMD in 38 patients, polymetastasis in 12 patients, PSA elevation in 5 patients, local recurrence in 2 patients, and regional recurrence in 1 patient.

**Figure 2:**
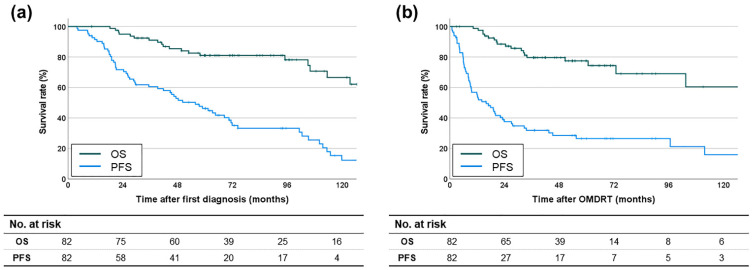
Kaplan-Meier survival curves for (**a**) OS and PFS rates after the first diagnosis of prostate cancer and (**b**) OS and PFS rates after OMDRT.

The OS and PFS rates following the initial prostate cancer diagnosis were as follows: the median OS was not reached, with 2-year and 5-year survival rates of 95% and 81.1%, respectively; the median PFS was 55.8 months, with 2-year and 5-year survival rates of 71.7% and 46.2%, respectively (Figure 2a). The OS and PFS rates following OMDRT, which were the primary endpoints of this study, were as follows: the median OS was not reached, with 2-year and 5-year survival rates of 88.5% and 77.5%, respectively; the median PFS was 16 months, with 2-year and 5-year survival rates of 37.6% and 26.5%, respectively (Figure 2b).

Univariate and multivariate analyses for PFS identified the type of OMD (synchronous vs. metachronous) and the timing of OMDRT (early vs. late) as significant independent prognostic factors. Synchronous OMD and early OMDRT were significantly associated with higher PFS (HR 0.537 [95% CI: 0.314–0.918, *p* = 0.023] and 0.461 [95% CI: 0.257–0.826, *p* = 0.009], respectively) (Table 2). For OS, univariate analysis indicated significant factors including age (HR 0.356, 95% CI: 0.128–0.992, *p* = 0.048), initial N stage (HR 0.399, 95% CI: 0.162–0.986, *p* = 0.046), timing of OMDRT (HR 0.214, 95% CI: 0.082–0.561, *p* = 0.002), and OMDRT modality (intensity-modulated RT (IMRT) vs. 3-dimensional conformal RT (3DCRT)) (HR 0.248, 95% CI: 0.088–0.704, *p* = 0.009). Multivariate analysis further confirmed that initial N stage (HR 0.285, 95% CI: 0.108–0.748, *p* = 0.011), timing of OMDRT (HR 0.219, 95% CI: 0.080–0.603, *p* = 0.003), and OMDRT modality (HR 0.238, 95% CI: 0.079–0.716, *p* = 0.011) were significant prognostic factors (Table 3).

**Figure 3:**
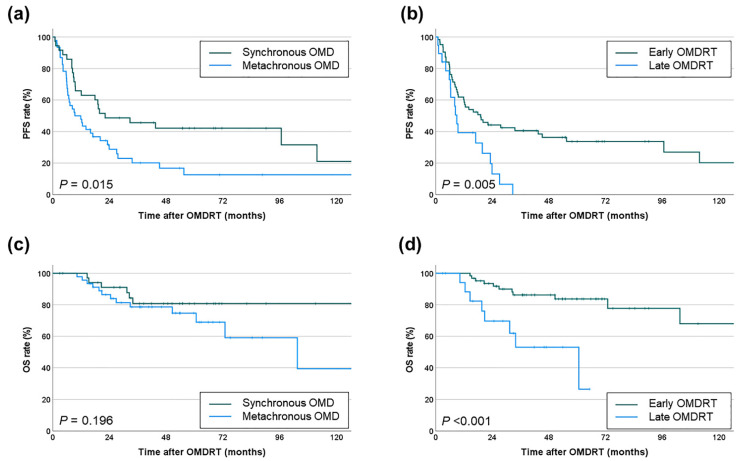
Comparison of (**a**) PFS rates by type of OMD and (**b**) PFS rates by timing of OMDRT; (**c**) OS rates by type of OMD and (**d**) OS rates by timing of OMDRT.

Kaplan-Meier analysis demonstrated that patients with synchronous OMD who underwent OMDRT had significantly higher PFS (median 22.2 months, 2-year 48.7%, 5-year 42.1%) compared to those with metachronous OMD (median 9.4 months, 2-year 28.8%, 5-year 12.6%, *p* = 0.015). However, there was no significant difference in OS (Figure 3a,c). Furthermore, patients who received early OMDRT exhibited significantly improved PFS (median 19.1 months, 2-year 44.2%, 5-year 33.7%) and OS (median not reached, 2-year 93.5%, 5-year 83.7%) compared to those who received late OMDRT (PFS: median 9.0 months, 2-year 13.1%, 5-year 0%, *p* = 0.005; OS: median 60.7 months, 2-year 69.7%, 5-year 53.1%, *p* < 0.001) (Figure 3b,d).

### 3.3. Subgroup Analysis

**Figure 4:**
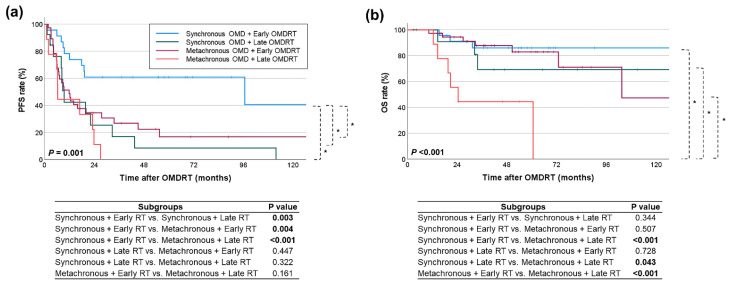
Comparison of (**a**) PFS and (**b**) OS rates across subgroups based on the type of OMD and the timing of OMDRT. Statistically significant comparison results are indicated in the figure with an asterisk (*) and bold font for the *p* values.

Subgroup analysis further elucidated the impact of OMDRT timing and OMD type on survival outcomes (Figure 4). PFS after OMDRT was highest in the synchronous OMD group treated with early OMDRT, with a median survival of 96.7 months and a 5-year survival rate of 60.9%. Conversely, the metachronous OMD group treated with late OMDRT experienced the lowest PFS, with a median survival of 6.4 months and a 5-year survival rate of 0% (*p* = 0.001). OS after OMDRT followed a similar pattern, with the lowest OS observed in the metachronous OMD group treated with late OMDRT (median 24.5 months, 5-year rate 44.4%) and the highest in the synchronous OMD group treated with early OMDRT (median not reached, 5-year rate 86%) (*p* < 0.001). Specifically, within the synchronous OMD group, patients who received early OMDRT had significantly higher PFS than those who received late OMDRT (*p* = 0.003). However, there was no significant difference in OS (*p* = 0.344). In the metachronous OMD group, early OMDRT was associated with significantly higher OS (*p* < 0.001) compared to late OMDRT, although there was no significant difference in PFS (*p* = 0.161).

**Figure 5:**
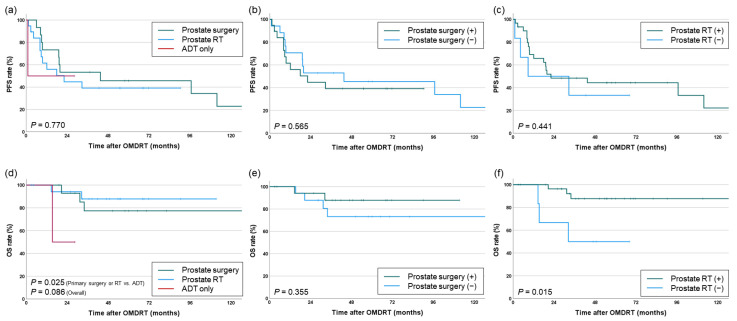
PFS (**a**–**c**) and OS (**d**–**f**) according to treatment approaches for the primary prostate in patients with synchronous OMD.

Within the synchronous OMD cohort, no significant differences in PFS were observed based on whether primary prostate treatment was performed (prostate surgery or RT vs. ADT: *p* = 0.748, overall: *p* = 0.770). There were also no differences based on whether prostate surgery or PPR was performed (*p* = 0.565 and 0.441, respectively). However, for OS, patients who received any form of primary prostate treatment had significantly higher survival rates (prostate surgery or RT vs. ADT: 5-year OS 83.1% vs. 50%, *p* = 0.025, overall: *p* = 0.770). In particular, the group that received PPR (before or concurrently with OMDRT) demonstrated significantly improved survival (5-year OS 87.7% vs. 50%, *p* = 0.015) (Figure 5).

## 4. Discussion

Our study provides a comprehensive analysis of the impact of OMDRT and PPR on survival outcomes in patients with OMPC. The findings highlight the significant prognostic value of the timing of OMDRT, with early OMDRT associated with improved PFS and/or OS not only in patients with synchronous OMD but also in those with metachronous OMD. Additionally, the study suggests a potential survival benefit from PPR, particularly in improving OS for patients with synchronous OMD. These results contribute to the growing body of evidence supporting the role of localized treatment strategies in the management of OMPC and underscore the need for further research to refine the optimal timing and combination of these therapies in this patient population.

The advent of next-generation imaging techniques for staging and restaging prostate cancer has significantly improved the detection of oligometastatic disease [22,23,24]. While systemic therapies such as ADT with or without androgen receptor pathway inhibitors remain the cornerstone of treatment [13,25,26,27,28], the relatively limited metastatic burden in these patients has spurred interest in MDT as a promising alternative. Both retrospective and prospective studies have demonstrated that MDT can enhance PFS and delay the initiation of systemic therapies in both hormone-sensitive and castration-resistant prostate cancer [16,17,18,27,29,30]. RT has been a key component of MDT strategies, despite variability across trials regarding radiation doses, target volumes, and the integration of systemic therapies [31,32].

Our study highlights the distinction between synchronous and metachronous OMD and its significant impact on treatment outcomes. While recent guidelines recommend categorizing de-novo OMD into these subtypes [22], their prognostic differences remain inadequately explored. Our findings reveal that patients with synchronous OMD had better PFS after OMDRT than those with metachronous OMD, despite no significant difference in OS. Synchronous OMD, which may exhibit more localized and less aggressive behavior, responded more favorably to early OMDRT, with a median PFS of 22.2 months and a 5-year PFS rate of 42.1%. In contrast, patients with metachronous OMD, particularly those receiving late OMDRT, demonstrated significantly poorer outcomes, with a median PFS of 6.4 months and no 5-year survivors. These results suggest that metachronous OMD may represent a more advanced disease stage, less responsive to localized therapies, highlighting the need for alternative or adjunctive systemic treatments. Further research is required to refine treatment strategies tailored to specific OMD types.

The timing of OMDRT is crucial in determining survival outcomes in OMPC. Our study aligns with the findings of the STOMP trial [16,33], which demonstrated that early MDT targeting all metastatic sites can significantly delay disease progression and prolong ADT-free survival. In our cohort, patients who received early OMDRT at the time of OMD diagnosis achieved a median PFS of 22.2 months and a 5-year PFS rate of 42.1%. In contrast, those who underwent late OMDRT had a markedly lower median PFS of 6.4 months, with no survivors reaching the 5-year milestone. These findings emphasize the substantial benefits of early OMDRT, particularly in patients with synchronous OMD, and highlight the importance of timely and strategically planned interventions to optimize disease control and improve long-term outcomes.

A comparison of our results with the ESTRO-ACROP Delphi consensus guidelines [21] reveals several key insights. The consensus emphasizes the importance of patient selection and the use of PSMA PET imaging for staging and restaging OMPC. Although our study employed advanced imaging techniques to categorize OMD and tailor RT strategies, the treatments were administered before the widespread adoption of PSMA PET. Nonetheless, our findings align with the consensus that metastasis-directed RT is effective across various oligometastatic settings, including both synchronous and metachronous OMD. Notably, our study underscores that early OMDRT, particularly when combined with aggressive treatment of the primary prostate in synchronous OMD patients, significantly improves survival outcomes. This highlights the potential benefits of timely and comprehensive local therapy. While the ESTRO-ACROP guidelines provide a framework for MDT, our study emphasizes the need for further research to optimize the timing and combination of RT modalities to maximize survival benefits in OMPC patients.

Our current study builds upon previous research, including our earlier publications [15,19,34,35,36,37], which have demonstrated the survival benefits of combining RT to the primary tumor with metastasis-directed RT in patients with metastatic cancer. Our earlier study [34] showed significant improvements in OS and biochemical failure-free survival with PPR in metastatic prostate cancer. In the present study, we specifically investigated the timing of OMDRT and PPR in the context of OMPC. We found that early OMDRT, particularly when combined with treatment targeting the primary tumor, has the potential to significantly improve survival outcomes, with a more pronounced benefit observed in OS compared to PFS. This may be explained by the “Seed and Soil” theory, which suggests that effective primary tumor treatment can influence the progression and treatment response of metastatic lesions [38,39]. Furthermore, it is possible that while early local interventions may not prevent progression at metastatic sites, they could delay subsequent disease-related deaths, thus improving OS. Based on these findings and supporting evidence from multiple studies [12,15,40,41], we propose that local RT targeting both the primary tumor and metastatic lesions represents an effective treatment strategy for patients with a low burden of metastatic prostate cancer. This underscores the importance of timely and advanced RT techniques and highlights the critical role of personalized RT strategies in improving survival in this patient population.

Despite the valuable insights provided by this study, several limitations must be acknowledged. First, the retrospective design introduces an inherent risk of selection bias, and the relatively small sample size may limit the broader applicability of the findings. The heterogeneity of the patient population, stemming from varied treatment approaches due to evolving institutional policies, likely contributed to the difficulty in conclusively demonstrating the survival benefit of PPR in patients with synchronous OMD. A key limitation is the absence of a control group receiving only systemic therapy, which restricts our ability to directly compare the impact of OMDRT and PPR against systemic treatments alone. Additionally, advancements in imaging technologies and RT techniques over the study period could have influenced outcomes. Although no statistically significant associations were found, using various hypofractionated RT dose schemes for OMDRT adds complexity. Moving forward, larger, prospectively designed studies are crucial, particularly those incorporating a control arm without OMDRT or PPR, to validate these findings and refine our understanding of the optimal timing and integration of OMDRT and PPR in various clinical scenarios. These future studies will be essential in clarifying the true survival benefit of these localized treatments.

## 5. Conclusions

In conclusion, this study highlights the potential survival benefits of early OMDRT in patients with OMPC, in both synchronous and metachronous cases. While our findings suggest that a timely and aggressive RT approach, possibly in combination with primary prostate treatment, could improve outcomes, additional data are needed to fully validate this conclusion. The study’s retrospective nature and the absence of a control group receiving systemic therapy alone underscore the need for further prospective studies to determine the impact of combined OMDRT and PPR on delaying disease progression and improving survival outcomes. Despite these limitations, our results provide valuable insights into the potential role of personalized RT strategies in managing OMPC.

## Figures and Tables

**Table 1:** Patients’ characteristics and treatment details (n = 82, all patients).

Variables		No.	%
Age (years)		median 66 (48–83)	
Initial T stage	T2	13	15.9
	T3	55	67.1
	T4	14	17.1
Initial N stage	N0	56	68.3
	N1	26	31.7
Initial M stage	M0	46	56.1
	M1	36	43.9
Initial PSA (ng/mL)		median 19.5 (1.4–1101.0)	
Initial GS		median 8 (6–10)	
Type of OMD	Synchronous OMD	36	43.9
	Metachronous OMD	46	56.1
Timing of OMDRT	Early OMDRT	60	73.2
	Late OMDRT	22	26.8
Patient subgroup by OMD type and OMDRT timing	Synchronous OMD + Early OMDRT	23	28.0
	Synchronous OMD + Late OMDRT	13	15.9
	Metachronous OMD + Early OMDRT	37	45.1
	Metachronous OMD + Late OMDRT	9	11.0
PSA level at OMDRT (ng/mL)		median 5.4 (0.0–1101.0)	
Number of OMDRT lesions	1	42	51.2
	2	15	18.3
	3	14	17.1
	4	4	4.9
	5	7	8.5
OMDRT sites	Bone	72	87.8
	Non-regional LN	9	11.0
	Lung	3	3.7
Field of OMDRT	Part of OMD lesions	6	7.3
	All OMD lesions	76	92.7
OMDRT, total dose (Gy)		median 45.0 (36.0–95.0)	
OMDRT, fractional dose (Gy)		median 3.8 (1.8–12.5)	
OMDRT modality	3DCRT	8	9.8
	IMRT	74	90.2
Prostate surgery	Performed	42	51.2
Prostate RT	Performed	71	86.6
Aim of prostate RT	Definitive	36	43.9
	Salvage	33	40.2
	Postoperative	2	2.4
Field of prostate RT	Prostate only	22	26.8
	Whole pelvis	48	58.5
	Unknown	1	1.2
Prostate RT, total dose (Gy)		median 57.6 (36.0–79.2)	
Prostate RT, fractional dose (Gy)		median 2.5 (1.8–3.4)	
Prostate RT modality	3DCRT	6	8.5
	IMRT	63	88.7
	Brachytherapy	2	2.8
Hormone therapy for OMD	Performed	78	95.1
	Concurrent with OMDRT	64	78.0

Abbreviations: OMD, Oligometastatic Disease; OMDRT, Oligometastasis-Directed Radiotherapy; PSA, Prostate-Specific Antigen; GS, Gleason Score; LN, Lymph Node; Gy, Gray (radiation dose); 3DCRT, Three-Dimensional Conformal Radiotherapy; IMRT, Intensity-Modulated Radiotherapy; RT, Radiotherapy; OMPC, Oligometastatic Prostate Cancer.

**Table 2:** Univariate and multivariate analysis for PFS in patients with oligometastatic prostate cancer treated with OMDRT.

Variables	UVA for PFS			MVA for PFS		
	HR	95% CI	*p* Value	HR	95% CI	*p* Value
Age (<66 vs. ≥66)	0.782	0.469–1.304	0.346			
Initial T stage	1.166	0.733–1.853	0.517			
Initial N stage	0.970	0.550–1.710	0.916			
Type of OMD (synchronous vs. metachronous)	0.519	0.304–0.887	0.016	0.537	0.314–0.918	0.023
Timing of OMDRT (early vs. late)	0.442	0.247–0.792	0.006	0.461	0.257–0.826	0.009
PSA level at OMDRT	0.997	0.992–1.002	0.234			
Number of OMDRT lesions	1.061	0.873–1.289	0.550			
OMDRT for bone	0.870	0.411–1.843	0.717			
OMDRT for non-regional LN	0.883	0.379–2.060	0.774			
OMDRT for lung	1.880	0.582–6.075	0.292			
Field of OMDRT	1.028	0.369–2.865	0.958			
OMDRT modality	0.534	0.252–1.134	0.102			
Prostate surgery	0.818	0.489–1.370	0.446			
Prostate RT	0.899	0.426–1.900	0.781			
Hormone therapy for OMD	2.662	0.798–8.886	0.111			
Hormone therapy concurrently with OMDRT	1.016	0.526–1.963	0.961			

Abbreviations: HR, Hazard Ratio; CI, Confidence Interval; OMDRT, Oligometastasis-Directed Radiotherapy; OMD, Oligometastatic Disease; PFS, Progression-free survival; PSA, Prostate-Specific Antigen; LN, Lymph Node; RT, Radiotherapy; MVA, Multivariate Analysis; UVA, Univariate Analysis.

**Table 3:** Univariate and multivariate analysis for OS in patients with oligometastatic prostate cancer treated with OMDRT.

Variables	UVA for OS			MVA for OS		
	HR	95% CI	*p* Value	HR	95% CI	*p* Value
Age (<66 vs. ≥66)	0.356	0.128–0.992	0.048	0.375	0.131–1.078	0.069
Initial T stage	1.494	0.632–3.529	0.360			
Initial N stage (N0 vs. N1)	0.399	0.162–0.986	0.046	0.285	0.108–0.748	0.011
Type of OMD (synchronous vs. metachronous)	0.533	0.202–1.407	0.204			
Timing of OMDRT (early vs. late)	0.214	0.082–0.561	0.002	0.219	0.080–0.603	0.003
PSA level at OMDRT	0.997	0.991–1.003	0.397			
Number of OMDRT lesions	1.241	0.902–1.707	0.186			
OMDRT for bone	2.373	0.314–17.948	0.403			
OMDRT for non-regional LN	0.450	0.060–3.377	0.437			
OMDRT for lung	0.046	0.000–1410.128	0.560			
Field of OMDRT	0.498	0.114–2.180	0.355			
OMDRT modality (IMRT vs. 3DCRT)	0.248	0.088–0.704	0.009	0.238	0.079–0.716	0.011
Prostate surgery	0.611	0.240–1.557	0.302			
Prostate RT	0.728	0.212–2.504	0.615			
Hormone therapy for OMD	3.558	0.425–29.797	0.242			
Hormone therapy concurrently with OMDRT	0.692	0.247–1.938	0.484			

Abbreviations: HR, Hazard Ratio; CI, Confidence Interval; OMDRT, Oligometastasis-Directed Radiotherapy; OS, Overall survival; OMD, Oligometastatic Disease; PSA, Prostate-Specific Antigen; LN, Lymph Node; RT, Radiotherapy; MVA, Multivariate Analysis; UVA, Univariate Analysis.

## Data Availability

The data presented in this study are available on request from the corresponding author.

## Supplementary Materials

The following supporting information can be downloaded at: https://www.mdpi.com/article/10.3390/cancers16183159/s1, Table S1. Comparison of baseline characteristics among subgroups based on OMDRT timing and OMD type.
