# Supplementary material for: The Role of Local Prostate and Metastasis-Directed Radiotherapy in the Treatment of Oligometastatic Prostate Cancer
Source: Cancers (Basel). 2024 Sep 14;16(18):3159. doi: 10.3390/cancers16183159 (PMC11429795; doi:10.3390/cancers16183159)
Supplement: Supplementary file 1 [file cancers-16-03159-s001.zip › cancers-3188522-supplementary.pdf]

Supplementary Table 1. Comparison of baseline characteristics among subgroups based on OMDRT timing and OMD type

Variables		Synchronous OMD + Early OMDRT	Synchronous OMD + Late OMDRT	Metachronous OMD + Early OMDRT	Metachronous OMD + Late OMDRT	*p* Value
Age (years)	<66 years	6 (26.1)	5 (38.5)	22 (59.5)	4 (44.4)	0.083
	≥66 years	17 (73.9)	8 (61.5)	15 (40.5)	5 (55.6)	
Initial T stage	T2	4 (17.4)	2 (15.4)	6 (16.2)	1 (11.1)	0.910
	T3	14 (60.9)	8 (61.5)	27 (73.0)	6 (66.7)	
	T4	5 (21.7)	3 (23.1)	4 (10.8)	2 (22.2)	
Initial N stage	N0	12 (52.2)	10 (76.9)	27 (73.0)	7 (77.8)	0.266
	N1	11 (47.8)	3 (23.1)	10 (27.0)	2 (22.2)	
Initial PSA (ng/mL)		105.2	24.7	51.2	24.2	0.334
PSA level at OMDRT (ng/mL)		104.7	9.2	8.2	6.6	0.025
Number of OMDRT lesions		2.26	2.54	1.59	2.33	0.056
OMDRT for bone	Yes	21 (91.3)	13 (100.0)	30 (81.1)	8 (88.9)	0.303
OMDRT for non-regional LN	Yes	2 (8.7)	1 (7.7)	4 (10.8/0	2 (22.2)	0.698
OMDRT for lung	Yes	0 (0.0)	0 (0.0)	3 (8.1)	0 (0.0)	0.285
Field of OMDRT	Part of OMD lesions	4 (17.4)	1 (7.7)	1 (2.7)	0 (0.0)	0.150
	All OMD lesions	19 (82.6)	12 (92.3)	36 (97.3)	9 (100.0)	
OMDRT modality	3DCRT	1 (4.3)	2 (15.4)	4 (10.8)	1 (11.1)	0.730
	IMRT	22 (95.7)	11 (84.6)	33 (89.2)	8 (88.9)	
Prostate surgery	Yes	11 (47.8)	8 (61.5)	19 (51.4)	4 (44.4)	0.843
Prostate RT	Yes	20 (87.0)	10 (76.9)	32 (86.5)	9 (100.0)	0.486
Hormone therapy for OMD	Yes	22 (95.7)	13 (100.0)	33 (89.2)	8 (88.9)	0.086
Hormone therapy concurrently with OMDRT	Yes	20 (87.0)	9 (69.2)	27 (75.0)	8 (88.9)	0.475

Abbreviations: 3DCRT, three-dimensional conformal radiation therapy; IMRT, intensity-modulated radiation therapy; LN, lymph node; OMD, oligometastatic disease; OMDRT, oligometastasis-directed radiotherapy; OMPC, oligometastatic prostate cancer; PSA, prostate-specific antigen; RT, radiotherapy
